# Comparison of the RADM2 and RACM chemical mechanisms in O_3_ simulations: effect of the photolysis rate constant

**DOI:** 10.1038/s41598-021-84629-4

**Published:** 2021-03-03

**Authors:** Chien-Hung Chen, Tu-Fu Chen, Shang-Ping Huang, Ken-Hui Chang

**Affiliations:** 1 Graduate School of Engineering Science and Technology, National Yunlin University of Science and Technology, Yunlin, Taiwan; 2 Department of Safety, Health and Environmental Engineering, National Yunlin University of Science and Technology, Yunlin, Taiwan

**Keywords:** Atmospheric chemistry, Environmental sciences, Environmental chemistry

## Abstract

Since the photolysis rate plays an important role in any photoreaction leading to compound sink and radical formation/destruction and eventually O_3_ formation, its impact on the simulated O_3_ concentration was evaluated in the present study. Both RADM2 and RACM were adopted with and without updated photolysis rate constants. The newly developed photolysis rates were determined based on two major absorption cross-section and quantum yield data sources. CMAQ in conjunction with meteorological MM5 and emission data retrieved from Taiwan and East Asia were employed to provide spatial and temporal O_3_ predictions over a one-week period in a three-level nested domain [from 81 km × 81 km in Domain 1 (East Asia) to 9 km × 9 km in Domain 3 (Taiwan)]. Four cases were analyzed, namely, RADM2, with the original photolysis rates applied in Case 1 as a reference case, RADM2, with the updated photolysis rates applied in Case 2, and RACM, with and without the updated photolysis rates applied in Cases 3 and 4, respectively. A comparison of the simulation and observed results indicates that both the application of updated photolysis rate constants and RACM instead of RADM2 enhanced all three error analysis indicators (unpaired peak prediction accuracy, mean normalized bias error and mean absolute normalized gross error). Specifically, RADM2 with the updated photolysis rates resulted in an increase of 12 ppb (10%) in the daily maximum O_3_ concentration in southwestern Taiwan, while RACM without the updated photolysis rates resulted in an increase of 20 ppb (17%) in the daily maximum O_3_ concentration in the same area. When RACM with the updated photolysis rate constants was applied in the air quality model, the difference in the daily maximum O_3_ concentration reached up to 30 ppb (25%). The implication of Case 4 (RACM with the updated photolysis rates) for the formation and degradation of α-pinene and d-limonene was examined.

## Introduction

The ozone concentration can be predicted through various air quality models with a specific photochemical mechanism, and the model results may support decision makers in policy formulation. Consequently, the simulation accuracy of air quality models is important. The results of an air quality model are affected by gaseous and aqueous phase chemical reactions, emissions, transport, deposition, topography and meteorological conditions. Atmospheric chemistry mechanisms play the most important role in atmospheric chemistry models^1^.

There are many photochemical mechanisms^2,3^, including the Regional Atmospheric Chemistry Mechanism (RACM^1^) and the Regional Acid Deposition Model, version 2 (RADM2^4^), for the prediction of the ambient O_3_ level. For example, RACM, developed by Stockwell et al.^1^, consists of 77 chemical species and considers 237 reactions, including 23 photolysis reactions that play an important role in many chemical reactions in the atmosphere. In particular, the revised RACM includes improvements to the mechanism of the oxidation of alkanes by hydroxyl radicals, ozonolysis of alkenes, reaction of alkenes with NO_3_ radicals, aromatic chemistry and, in particular, chemistry of isoprene and terpenes^1^. The Regional Atmospheric Chemistry Mechanism, version 2 (RACM2), created by Goliff et al.^5^, includes updated reaction schemes, rate constants and product yields on the basis of RACM.

The simulation results of various box models and 3-D air quality models have been considered to compare the O_3_ simulation differences among various chemical mechanisms^3,6–9^. For example, Cross and Stockwell^6^ evaluated the EMEP^10^ (Cooperative Program for the Monitoring and Evaluation of the Long-Range Transmission of Air Pollutants in Europe), RADM2 and RACM mechanisms and found that RADM2 yielded the lowest O_3_ levels; the differences in O_3_ precursors among the various mechanisms were insignificant under clean conditions and more profound under polluted conditions. Although similar simulated O_3_ concentrations have been reported among several mechanisms, including RADM2, RACM, RACM2, CB05^11^ (2005 Carbon Bond Mechanism), SAPRC99^12^ (Statewide Air Pollution Research Center), and CB4^13^ (Carbon Bond 4), certain mechanisms better predict the peak O_3_ level, and different models exhibit significant differences over many urban areas^7^. Furthermore, the model performance also depends on the O_3_ level, e.g., CB05 attains the best performance at observed O_3_ levels > 75 ppb, while CB4 yields better results at O_3_ < 75 ppb^8^. Polluted air may be dominated by reactive nitrogen chemistry^14^; hence, the latter explains the different model performance results. Sarwar et al.^15^ incorporated RACM2 into CMAQ^16^ (Community Multiscale Air Quality modeling system) for a comparison to CB05TU (CB05 with updated toluene chemistry). The results revealed that RACM2 increased the monthly mean sulfate by 10%, nitrate by 6%, ammonium by 10%, and anthropogenic secondary organic aerosols by 42%. The increased inorganic and organic aerosol levels obtained with RACM2 agreed better with observed data. Jimenez et al.^3^ evaluated different photochemical mechanisms without a comparison to observed O_3_ concentrations. In summary, the performance of different chemistry mechanisms may be site-specific (e.g., VOC- or NOx-sensitive areas and/or polluted or clean conditions) and scenario-specific.

Photolysis reactions are essential to the atmospheric chemistry^17^. Accurate photolysis rate estimates, therefore, must be obtained to reasonably predict the effects of air pollution. The photolysis rate of each species, mainly influenced by the absorption cross-section and quantum yield, which are functions of the wavelength, is partly responsible for compound sink establishment^18^ and radical formation/destruction^19,20^. In fact, HCHO photolysis has been found to be the most important source of OH radicals, followed by O_3_ and nitrous acid (HONO)^21,22^. Since OH radicals and aldehydes are the principal products of the near-IR photolysis of peroxyl radicals (RO2), there exists a clear need to better estimate the photolysis rate of RO2^23^. Through uncertainty analysis, Chen and Brune^24^ found that photolysis via HONO and OH radical reactions with aldehydes and NO_2_ contributes to O_3_ production. Hanna et al.^25^ evaluated model input variables (emissions, initial and boundary conditions, meteorological variables, and chemical reactions) and concluded that the uncertainties in ozone prediction were most strongly correlated with the uncertainties in the NO_2_ photolysis rate. Hence, the effect of the photolysis rate on the simulated O_3_ level with any photochemical mechanism is expected. The NO_2_ photolysis and ozone production rates in the troposphere are also affected by aerosols. He and Carmichael^26^ pointed out that ozone production may be either enhanced or weakened in the upper troposphere, depending on the scattering and absorption capacity of aerosols and the availability of NOx, whereas aerosol particles decrease the NO_2_ photolysis rate and reduce ozone production in the lower troposphere. Wang et al.^27^ concluded that aerosols led to a decrease of 24% and 30% in the seasonal mean NO_2_ photolysis rate in summer and winter, respectively, based on photolysis frequency measurement during the 2012–2015 period in Beijing, and the monthly mean daytime net ozone production rate decreased by up to 25% due to the light extinction effect of aerosols, according to an observation campaign in August 2012.

Many studies on photolysis rates have been published, and more up-to-date data have become available. The impact of the application of updated photolysis rates on the simulation results for various chemical mechanisms is an important topic to improve the performance of air quality models. This study was undertaken to compare the differences in simulated ozone concentration between the RADM2 and RACM mechanisms with and without newly updated photolysis rate constants through CMAQ. The case with the smallest errors was further applied to simulate API (α-pinene) and LIM (d-limonene) levels, since they constitute the major biogenic revisions in RACM.

## Methods

Based on the application of RADM2 in the CMAQ model, the gas-phase chemical mechanism was incorporated to establish RACM. In addition, new parameters of the photolysis rate were also determined to update both RADM2 and RACM in this study.

The air quality modeling system consists of 3 networks: (1) meteorological, (2) emission and (3) CMAQ. Meteorological data were generated with the Fifth-Generation NCAR/Penn State Mesoscale Model (MM5^28^), and emission data retrieved from East Asia and Taiwan were incorporated into the emission system. The CMAQ model with multilevel nested domains has been applied to examine various issues in Taiwan, such as the direct and indirect effects of long-range transport^29^, cause analysis of serious air pollution events^30^, effectiveness assessment of emissions reduction strategies^31^, and simulation of emissions reduction to achieve air quality targets^32^. Newly developed photolysis rates were then processed via the CMAQ Photolysis Rate Processor and incorporated into CMAQ. A detailed description of the air modeling system is provided in Sec. 2.3.

### Establishment of RACM

Compared to RADM2, the number of chemical reactions in RACM is highly increased, particularly in regard to aromatic chemistry^1^. Notably, VOCs are grouped into 16 anthropogenic and three biogenic sources. The oxidation of isoprene, API and LIM is also addressed in detail. Chemical reactions can be divided into two parts: photolysis and nonphotolysis reaction parts. Various types of reactions, reactants, products and their coefficients must be incorporated.

### Update of photolysis rate constants

Two major data sources of the absorption cross-section and quantum yield are available to update the photolysis rate constants in the present study. One data source was published by the JPL^33^, and the other data source was the online search system (http://www.atmosphere.mpg.de/enid/2.html) of the Atmospheric Chemistry Department of the Max Planck Institute. Most of the rate constants of photolytic species may be updated in RADM2 and RACM. A list of data sources of the absorption cross-section, wavelength range and quantum yield before and after the update are summarized in Table 1 for all the above 23 photolysis reactions. The reaction numbers in the first column of Table 1 correspond to the photoreactions listed in Table 2. After the updated data were incorporated, differences in the wavelength range were immediately observed. The updated wavelength ranges of most species increased, whereas those of certain species decreased. For example, the wavelength range of NO_3_ hardly changed (402–695 nm before adjustment vs. 403–692 nm after adjustment), while that of NO_2_ notably changed (185–427 vs. 242–665 nm).

**Table 1:** List of the changes in wavelength, cross-section and quantum yield before and after updating.

Reaction number^a^	Species^c^	Before updating			After updating		
		Cross-section (σ, cm^2^ molecule^−1^)		Quantum yield (ϕ)	Cross-section (σ, cm^2^ molecule^−1^)		Quantum yield (ϕ)
		Wavelength range (λ, nm)	References	References	Wavelength range (λ, nm)	References	References
1	NO_2_	185–427	Bass et al.^54^	Gardner et al.^55^	242–665	JPL^33^	Troe^56^
2	O_3_	185–735	JPL^57^	Moortgat and Kudzus^58^	185–830	JPL^33^	JPL^59^
3	O_3_	185–735	JPL^57^	Stockwell et al.^4^	185–830	JPL^33^	Stockwell et al.^1^
4	HONO	310–392	Stockwell and Calvert^60^	Stockwell et al.^4^	184–397	JPL^33^	JPL^59^
5	HNO_3_	190–327	Molina and Molina^61^	Stockwell et al.^4^	190–352	JPL^33^	Stockwell et al.^1^
6	HNO_4_	188–332	Molina and Molina^61^	Stockwell et al.^4^	190–352	JPL^33^	Stockwell et al.^1^
7	NO_3_	402–695	Graham and Johnston^62^	Magnotta and Johnson^63^	403–692	JPL^33^	Johnston et al.^64^
8	NO_3_	402–695	Graham and Johnston^62^	Magnotta and Johnson^63^	403–692	JPL^33^	Johnston et al.^64^
9	H_2_O_2_	190–352	Lin et al.^65^	Stockwell et al.^4^	190–355	JPL^33^	Stockwell et al.^1^
10	HCHO	246–367	Moortgat et al.^66,67^	Moortgat et al.^67^	226–375	JPL^33^	Smith et al.^68^
11	HCHO	246–367	Moortgat et al.^66,67^	Moortgat et al.^67^	226–375	JPL^33^	Moortgat et al.^67^
12	ALD	206–352	Calvert and Pitts^69^	Meyrahn et al.^70^	202–361	JPL^33^	Atkinson et al.^71^
13	OP1	210–357	Molina and Arguello^72^	Stockwell et al.^4^	210–370	JPL^33^	JPL^59^
14	OP2	210–357	Molina and Arguello^72^	Stockwell et al.^4^	210–370	JPL^33^	JPL^59^
15	PAA	190–352	Giguere and Olmos^73^	Stockwell et al.^4^	190–355	JPL^33^	Stockwell et al.^1^
16	KET	277–322	Calvert and Pitts^69^	Gardner et al.^55^	202–355	Martinez et al.^74^	Gardner et al.^55^
17	GLY	232–457	Plum et al.^75^	Carter et al.^76^	232–526	JPL^33^	Atkinson et al.^77^
18	GLY	232–457	Plum et al.^75^	Carter et al.^76^	232–526	JPL^33^	Atkinson et al.^77^
19	MGLY	232–457	Plum et al.^75^	Carter et al.^76^	200–493	JPL^33^	Carter et al.^76^
20	DCB	185–362	Carter et al.^76^	Carter et al.^76^	185–362	Carter et al.^76^	Carter et al.^76^
21	ONIT	263–327	Calvert and Pitts^69^	Stockwell et al.^4^	270–330	Atkinson et al.^71^	Atkinson et al.^71^
22^b^	MACR	226–380	Gardner et al.^55^	Gardner et al.^55^	250–395	JPL^33^	Gierczak et al.^78^
23^b^	HKET	277–322	Calvert and Pitts^69^	Gardner et al.^55^	202–355	Martinez et al.^74^	Gardner et al.^55^

^a^The reaction number corresponds to the photolysis reaction in Stockwell et al.^1^.

^b^Reactions are only applied in RACM^1^.

^c^Please refer to Table 2 for the abbreviations.

**Table 2:** RACM 23 photolysis reactions.

Reaction no	Reaction	Definition
1	NO_2_ → O^3^P + NO	
2	O_3_ → O^1^D + O_2_	
3	O_3_ → O^3^P + O_2_	
4	HONO → HO + NO	
5	HNO_3_ → HO + NO_2_	
6	HNO_4_ → 0.65HO_2_ + 0.65NO_2_ + 0.35HO + 0.35NO_3_	
7	NO_3_ → NO + O_2_	
8	NO_3_ → NO_2_ + O^3^P	
9	H_2_O_2_ → HO + HO	
10	HCHO → H_2_ + CO	HCHO: formaldehyde
11	HCHO → 2HO_2_ + CO	
12	ALD → MO_2_ + HO_2_ + CO	ALD: acetaldehyde and higher aldehydes
13	OP1 → HCHO + HO_2_ + HO	OP1: methyl hydrogen peroxide
14	OP2 → ALD + HO_2_ + HO	OP2: higher organic peroxide
15	PAA → MO_2_ + HO	PAA: peroxyacetic acid and higher analogs
16	KET → ETHP + ACO_3_	KET: ketones
17	GLY → 0.13HCHO + 1.87CO + 0.87H_2_	GLY: glyoxal
18	GLY → 0.45HCHO + 1.55CO + 0.80HO_2_	
19	MGLY → CO + HO_2_ + ACO_3_	MGLY: methylglyoxal and other α-carbonyl
20	DCB → TCO_3_ + HO_2_	DCB: unsaturated dicarbonyls
21	ONIT → 0.2ALD + 0.8KET + HO_2_ + NO_2_	ONIT: organic nitrate
22	MACR → CO + HCHO + HO_2_ + ACO_3_	MACR: methacrolein and other unsaturated compounds
23	HKET → HCHO + HO_2_ + ACO_3_	HKET: hydroxy ketone

The photolysis rate coefficient (J value, s^−1^ nm^−1^) was calculated via the integration of the products of the absorption cross-section [(σ(λ)], photodissociation quantum yield [(φ(λ)] and actinic flux [(F(λ)], which are all related to the wavelength as:

1 $$ J(\lambda ) = \int\limits_{\lambda } {F\left( \lambda \right)\sigma \left( \lambda \right)\varphi \left( \lambda \right)d\lambda } $$

where *F*(λ) is expressed in photons cm^−2^ s^−1^ nm^−1^, σ(λ) is expressed in cm^2^ molecule^−1^ and φ(λ) varies between 0 and 1. Integration of the area yields the rate constant expressed in s^−1^.

**Figure 1:**
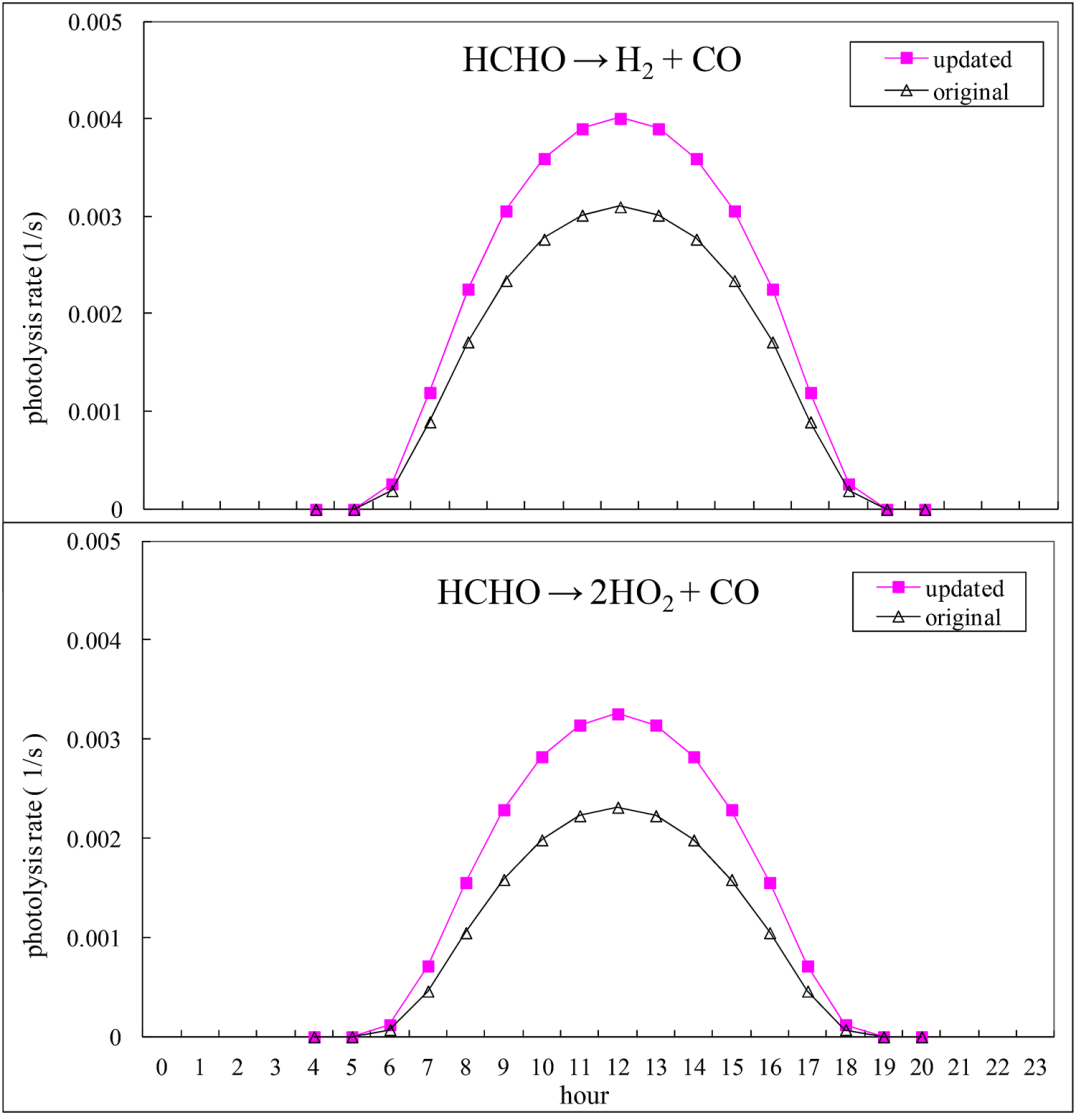
Comparison of the original and adjusted diurnal variations in the HCHO photolysis rate.

HCHO was adopted as an example to illustrate the calculation steps for the determination of the photolysis rate constant, since it is one of the major species responsible for OH radical generation^15^. The modification of σ(λ) and φ(λ) in HCHO is tabulated in Table 3 for data input into Eq. (1). The calculated photolysis rate constant resulting from the integration of Eq. (1) for HCHO is shown in Fig. 1 for a given day. Clearly, the difference between these two cases of the original and updated diurnal photolysis rate coefficients is remarkable. At noon, the updated photolysis rate exhibits a value more than 50% higher than the original photolysis rate. Hence, the effect on the subsequent simulated O_3_ level may be profound. The results for other important species, such as NO_2_, O_3_, and HONO, are provided in the Supplemental Information.

**Table 3:** Modification of the wavelength (λ), cross-section (σ) and quantum yield (ϕ) for HCHO.

Original in RADM2				Updated in this study			
λ	σ	ϕ		λ	σ	ϕ	
(nm)	10^20^ (cm^2^ molecule^−1^)	Φ1^a^	Φ2^b^	(nm)	10^20^ (cm^2^ molecule^−1^)	Φ1^a^	Φ2^b^
246	0.00	0.0077	0.0057	226	0.02	0	0
250	0.04	0.48	0.34	228	0.02	0	0
253	0.12	0.49	0.32	231	0.03	0	0
256	0.28	0.49	0.32	233	0.03	0	0
259	0.51	0.50	0.32	236	0.06	0	0
263	0.55	0.49	0.33	239	0.07	0	0
266	0.93	0.48	0.36	242	0.13	0	0
270	1.16	0.46	0.41	245	0.14	0	0
273	1.60	0.39	0.46	248	0.25	0	0
277	1.58	0.34	0.52	248	0.25	0	0
281	2.27	0.33	0.61	251	0.27	0.49	0.31
285	2.13	0.31	0.68	254	0.45	0.50	0.3
289	2.26	0.29	0.72	258	0.48	0.49	0.31
294	2.99	0.27	0.74	261	0.70	0.47	0.33
298	1.52	0.25	0.75	264	0.74	0.45	0.36
302	2.28	0.25	0.75	268	1.13	0.43	0.41
303	6.33	0.25	0.75	272	1.30	0.40	0.45
304	4.67	0.25	0.75	275	1.84	0.37	0.51
305	4.50	0.25	0.75	279	1.86	0.34	0.56
306	2.04	0.25	0.75	283	2.55	0.32	0.62
300	1.41	0.25	0.75	287	2.33	0.29	0.67
308	2.96	0.26	0.75	292	2.66	0.27	0.71
309	1.75	0.26	0.75	296	3.28	0.25	0.74
310	0.73	0.26	0.75	300	1.60	0.24	0.76
311	1.34	0.27	0.74	305	4.42	0.24	0.76
312	1.25	0.27	0.73	310	1.63	0.26	0.74
313	3.92	0.28	0.72	315	4.09	0.32	0.69
314	3.95	0.31	0.69	320	1.53	0.40	0.6
317	1.53	0.39	0.59	325	2.79	0.51	0.49
322	2.11	0.51	0.46	330	1.99	0.66	0.34
327	1.92	0.68	0.31	335	0.20	0.74	0.17
332	0.21	0.76	0.12	340	2.39	0.65	0
337	1.63	0.64	0.0034	345	0.76	0.50	0
342	0.67	0.50	0	350	0.19	0.38	0
347	0.15	0.37	0	355	0.96	0.22	0
352	0.72	0.23	0	360	0.01	0.0040	0
357	0.0091	0.10	0	365	0.01	0	0
362	0	0.0059	0	370	0.04	0	0
367	0	0		375	0.00	0	0

^a^Φ1: HCHO + hv → H_2_ + CO.

^b^Φ2: HCHO + hv → 2HO_2_ + CO.

Via the incorporation of the updated photolysis rate coefficients for these 23 reactions, the accuracy of O_3_ simulation may be enhanced.

### Air quality modeling

**Figure 2:**
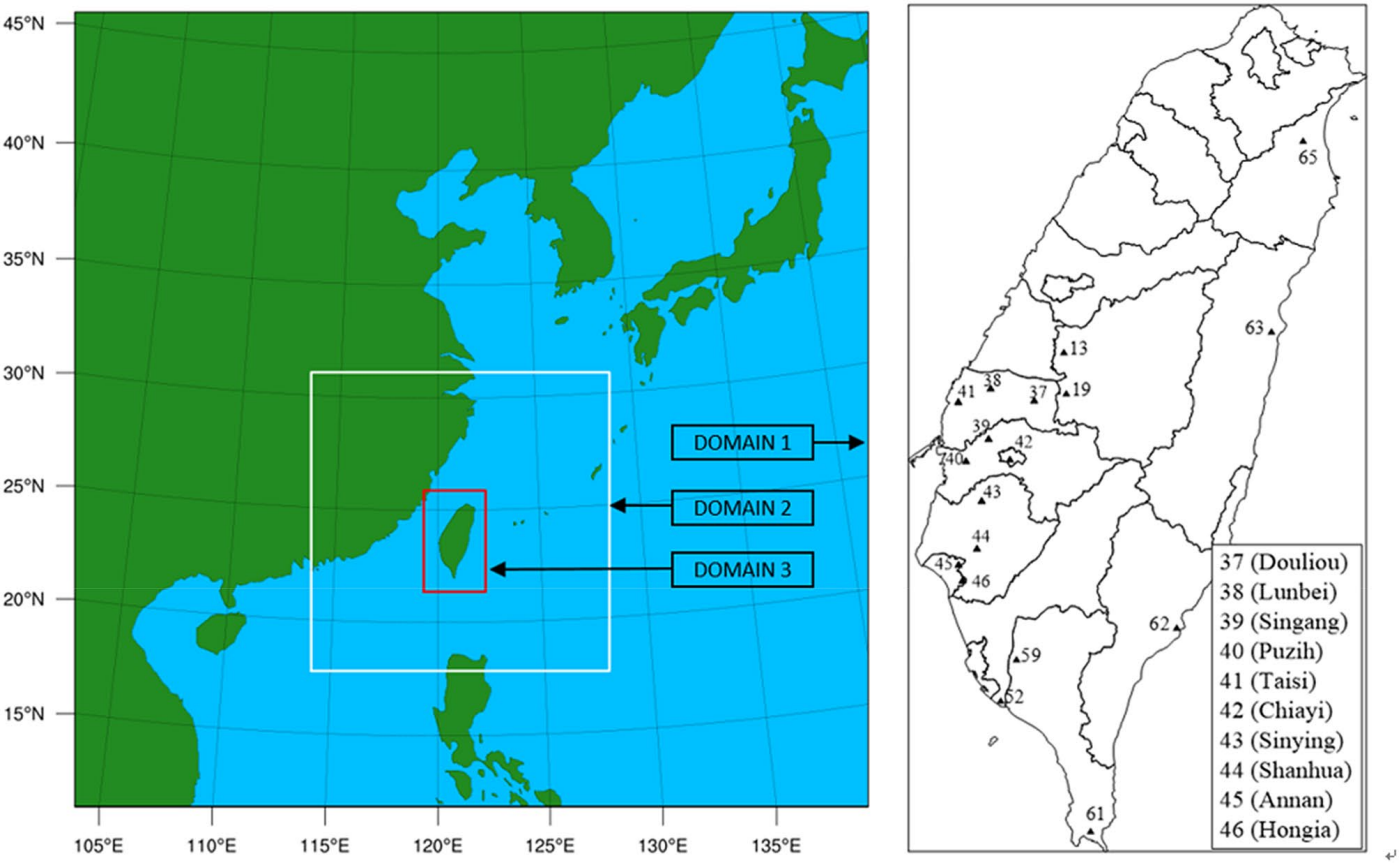
Configuration of the three-level nested domain used in this study and the monitoring stations in Domain 3 (the left map is produced with NCAR Command Language Version 6.6.2, and the right map is plotted with Surfer(R) Version 8.05, https://www.goldensoftware.com/products/surfer).

The configuration of the three-level nested simulation domain is shown in Fig. 2, including East Asia in Domain 1 with an 81 km × 81 km resolution, Southeast China and Taiwan in Domain 2 with a 27 km × 27 km resolution, and the entirety of Taiwan in Domain 3 with a 9 km × 9 km resolution. The meteorological field was calculated with MM5, which has already been extensively tested and adopted to investigate various issues in Taiwan. For example, Lin et al.^34^ constructed an MM5-based model to study and analyze the impact of the heat island effect on regional weather conditions in Taiwan, and Chien et al.^35^ performed an evaluation study with a real-time MM5 mesoscale ensemble prediction system during the rainy season. The ozone episode from May 22 to 29, 2003, was selected for simulation in this study because observed ozone and precursor data are available.

Holnicki and Nahorski^36^ demonstrated that the spatial variability in the simulation uncertainty highly depends not only on the category of the emission source but also on the contributing emission sources and their quantity. The first version of the Taiwan emission inventory dataset, called the Taiwan Emission Dataset System version 1 (TEDS 1), was established for the base year of 1988, after which TEDS has been updated every three years with more elaborate categories and more precise emission source amounts. Consequently, TEDS 6 with a base year of 2003 containing a detailed data generation description^37^ was adopted in this study in regard to anthropogenic emissions, and the Taiwan Biogenic Emission Inventory System (TBEIS 2)^38,39^ was applied to estimate the biogenic emissions in Taiwan. Regarding the other regions in East Asia, anthropogenic emission simulation mainly adopted the inventory dataset of the Regional Emission Inventory in Asia^40^, and the quantity of emissions was distributed at a 1 km × 1 km resolution based on the population distribution in East Asia at the same resolution. The East Asia Biogenic Emission Inventory System^41^ with a high resolution (1 km × 1 km) was applied to the simulation of the biogenic emissions originating from East Asia, excluding Taiwan.

### Description of the simulation cases

To examine RADM2 and RACM and the influence of the updated photolysis rates, four simulation cases were designed, as listed in Table 4. Chemical reaction mechanism RADM2 and the original photolysis rates were applied in Case 1 as a reference case. RADM2 with the updated photolysis rates was adopted in Case 2 in this study. Chemical reaction mechanism RACM and the original photolysis rates were applied in Case 3, and RACM with the updated photolysis rates was applied in Case 4. Thus, Cases 1 and 3 serve as benchmarks for RADM2 and RACM, respectively, and the difference between Cases 3 and 1 represents the net effect of RACM. The resulting differences between Cases 2 and 1 and between Cases 4 and 3 represent the effect of the updated photolysis rates. Finally, the difference in O_3_ concentration between Cases 4 and 1 indicates the overall impact of both the new RACM version and the updated photolysis rates.

**Table 4:** Four simulation cases.

Simulation case	Chemistry mechanism	Photolysis rate
Case 1	RADM2	Original data in RADM2
Case 2	RADM2	Updated photolysis rates
Case 3	RACM	Original data in RACM
Case 4	RACM	Updated photolysis rates

## Results and discussion

Case 1 was adopted as the reference case and compared to the other cases, and the influences of RACM and the updated photolysis rate constants on the model simulation results were analyzed.

### Comparison of the observed and simulated ozone concentrations

The period from May 24 to 29 was selected for the simulation and analysis in this study due to the presence of high-O_3_ pollution events. In general, the eastward wind blowing originating from the Pacific Ocean in spring is affected by the central mountain range of Taiwan. The southern portion of the easterly wind rounds the mountains, reaches the coast of southwestern Taiwan or the sea nearby, and then establishes local circulation conditions in this area. Moreover, the air mass near the inland surface follows the above local circulation conditions, is transported northward to mountains, accumulates along the low-elevation mountains occurring from southwestern to central Taiwan and generates high O_3_ concentrations in these areas.

**Figure 3:**
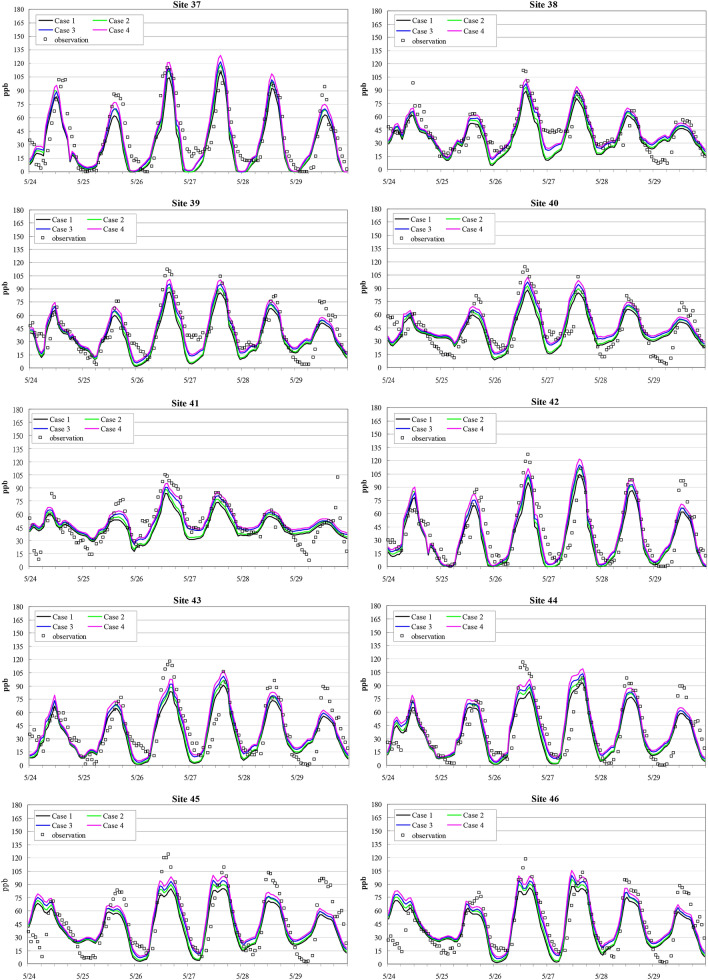
Time series of the simulated and observed ozone concentrations in Cases 1 to 4 at the different monitoring stations located in southern Taiwan during the 6-d period from May 24 to 29, 2003.

A comparison of the observed and simulated ozone concentrations in the above four cases from May 24 to 29 around central and southern Taiwan (Domain 3) is shown in Fig. 3. The daily maximum O_3_ concentration usually occurred at approximately 2 pm in suburban areas. In general, all simulated data suitably matched the observed O_3_ concentrations with a few exceptions. Nevertheless, certain differences in the simulated peak ozone concentration were observed among these four cases. Examining the resulting differences between Cases 1 and 2, the diurnal pattern of the simulated ozone concentration was consistent, and the only difference was the high peaks observed during the daytime in Case 2. The ozone concentrations at night in Cases 3 and 4 were consistently higher than those in Cases 1 and 2. These differences may result from the additional reactions regarding peroxyl radicals in RACM because NO_3_ is a critical reactive species at night but is quickly decomposed by sunlight^42,43^. Accordingly, updating photolysis rate data does play an important role in the formation of ozone peaks. A comparison of these four cases further indicates that the simulated ozone concentrations in Case 4 were always higher than those in Case 1, apparently due to the updated photolysis rates, while the increase at night was influenced by RACM instead of RADM2.

An error comparison based on the unpaired peak prediction accuracy (UPPA), mean normalized bias error (MNBE), and mean absolute normalized gross error (MANGE) of the hourly observed values at O_3_ > 30 ppb among the above four cases at 10 selected stations in southwestern Taiwan (Fig. 2, insert) is presented in Table 5 with the equations shown below:

2 $$ UPPA = \frac{1}{N}\sum\limits_{i = 1}^{N} {\frac{{C_{p} \left( {i,t} \right)_{\max } - C_{o} \left( {i,t} \right)_{\max } }}{{C_{o} \left( {i,t} \right)_{\max } }}} $$

3 $$ MNBE = \frac{1}{24N}\sum\limits_{i = 1}^{N} {\frac{{\left\{ {C_{p} \left( {i,t} \right) - C_{o} \left( {i,t} \right)} \right\}}}{{C_{o} \left( {i,t} \right)}}} ,\begin{array}{*{20}c} {} & {t = 1,\;24} \\ \end{array} $$

4 $$ MANGE = \frac{1}{24N}\sum\limits_{i = 1}^{N} {\frac{{\left| {C_{p} \left( {i,t} \right) - C_{o} \left( {i,t} \right)} \right|}}{{C_{o} \left( {i,t} \right)}}} ,\begin{array}{*{20}c} {} & {t = 1,\;24} \\ \end{array} $$

**Table 5:** Error analysis of the simulated O_3_ levels at 10 sites during the 6-d period from May 24 to 29, 2003.

Site number	Average O_3_ concentration (ppb)					UPPA^a^ (%)				MNBE^b^ (%)				MANGE^c^ (%)			
	Observed^d^	Case 1	Case 2	Case 3	Case 4	Case 1	Case 2	Case 3	Case 4	Case 1	Case 2	Case 3	Case 4	Case 1	Case 2	Case 3	Case 4
37	40	31	35	37	40	− 16	− 8	− 7	− 0.3	− 24	− 16	− 13	− 5.0	35	32	30	30
38	43	37	39	43	45	− 18	− 13	− 10	− 5.6	− 19	− 13	− 6	− 1.1	24	22	17	16
39	43	35	38	40	43	− 19	− 14	− 12	− 7.3	− 22	− 16	− 11	− 4.7	29	26	22	21
40	45	42	44	47	50	− 20	− 16	− 13	− 8.8	− 15	− 9	− 2.4	2.8	25	23	20	20
41	50	45	47	51	53	− 26	− 22	− 19	− 14.8	− 14	− 10	− 2.3	2.4	19	16	13	13
42	40	32	35	37	40	− 13	− 6	− 6	0.8	− 21	− 13	− 10	− 1.9	33	31	28	28
43	42	35	38	40	43	− 19	− 14	− 12	− 6.8	− 23	− 16	− 11	− 5.4	30	27	24	23
44	40	36	39	42	45	− 18	− 13	− 11	− 5.3	− 16	− 9	− 4.3	2.4	27	24	22	23
45	48	42	45	48	51	− 26	− 22	− 20	− 15.7	− 19	− 13	− 8.4	− 3.2	29	27	24	23
46	46	42	45	48	51	− 18	− 12	− 10	− 5.0	− 16	− 10	− 5.3	0.3	24	22	20	19
AVG	44	38	41	43	46	− 19	− 14	− 12	− 6.9	− 19	− 13	− 7.3	− 1.3	27	25	22	22

^a^UPPA (unpaired peak prediction accuracy): $UPPA = \frac{1}{N}\sum\limits_{i = 1}^{N} {\frac{{C_{p} \left( {i,t} \right)_{\max } - C_{o} \left( {i,t} \right)_{\max } }}{{C_{o} \left( {i,t} \right)_{\max } }}}$

^b^MNBE (mean normalized bias error): $$MNBE = \frac{1}{24N}\sum\limits_{i = 1}^{N} {\frac{{\left\{ {C_{p} \left( {i,t} \right) - C_{o} \left( {i,t} \right)} \right\}}}{{C_{o} \left( {i,t} \right)}}} ,\begin{array}{*{20}c} {} & {t = 1,\;24} \\ \end{array}$$

^c^MANGE (mean absolute normalized gross error): $$MANGE = \frac{1}{24N}\sum\limits_{i = 1}^{N} {\frac{{\left| {C_{p} \left( {i,t} \right) - C_{o} \left( {i,t} \right)} \right|}}{{C_{o} \left( {i,t} \right)}}} ,\begin{array}{*{20}c} {} & {t = 1,\;24} \\ \end{array}$$

^d^Average O_3_ concentration during the 6-d period from May 24 to 29, 2003.

where $C_{p} \left( {i,t} \right)$ is the predicted value on day *i* during hour *t*, $C_{o} \left( {i,t} \right)$ is the observed value on day *i* during hour *t*, N is the number of days (*N* = 6 for the 6-day simulation period), and $C_{p} \left( {i,t} \right)_{\max }$ and $C_{o} \left( {i,t} \right)_{\max }$ are the predicted and observed maximum 1-h O_3_ concentrations, respectively, on day *i*.

UPPA essentially represents the bias in the 1-h O_3_ peak concentration. MNBE is a useful model performance indicator because it avoids overinflation of the observed value range, especially at low concentrations^44^. MANGE quantifies the mean absolute deviation in the residuals and is a robust measure of the overall model performance, thus providing a useful basis for the comparison of model simulations across different model grids or episodes^44^. Negative values were found in all four cases, indicating that the simulated ozone concentrations were underestimated. The magnitudes of UPPA, MNBE and MANGE all differed at any given station in the same simulation case, e.g., the absolute UPPA value was the highest at one station, while the highest MNBE and MANGE values occurred at the same, yet different, station. The mean UPPA and MNBE values at all 10 stations in Case 1 were approximately − 19%. However, they decreased in Cases 2, 3 and 4, with a UPPA value of only − 6.9% in Case 4. Comparing Cases 1 and 3, all three error indicators decreased after RADM2 was replaced with RACM, namely, the UPPA, MNBE and MANGE values decreased from − 19% to − 12%, − 19% to − 7.3%, and 27% to 22%, respectively. This clearly indicates a better match obtained between the observed O_3_ levels and the simulated RACM data. Similarly, error analysis also demonstrated improvement when the updated photolysis rate constants were applied (Case 2 vs. 1 and Case 4 vs. 3). This confirms the important role of photolysis in the overall atmospheric chemistry with respect to O_3_ simulation. Finally, the error analysis results indicated that MANGE in all four cases occurred within the benchmark range used to evaluate the model performance^45,46^, e.g., MANGE ranged from 30 to 35%. All error data in Cases 3 and 4 occurred within the benchmark ranges of UPPA and MNBE, i.e., UPPA ranged from ± 15 to ± 20%, and MNBE ranged from ± 5 to ± 15%.

Compared to the performance evaluation results reported by Yu et al.^8^ for three chemical mechanisms, i.e., CB4, CB5 and SAPRC, with NMBE ranging from 11 to 28% for O_3_, the simulated O_3_ concentrations with RADM2 and RACM in this study tended to be underestimated with MNBE ranging from − 19 to − 7%. Underestimation of the simulated O_3_ level mainly occurred during the daytime and might be due to the underestimation of the VOC concentration because of the nature of the VOC limitations in southern Taiwan^47^.

In summary, Case 4 yields the smallest errors across all three indicators, i.e., UPPA, MNBE and MANGE. This demonstrates a good utilization of RACM with the newly developed photolysis data. The validity of the simulated results should provide a reasonable assurance for the simulated O_3_ results, which is further examined below.

### Influence of the chemical mechanisms (comparison of Case 3 vs. 1)

**Figure 4:**
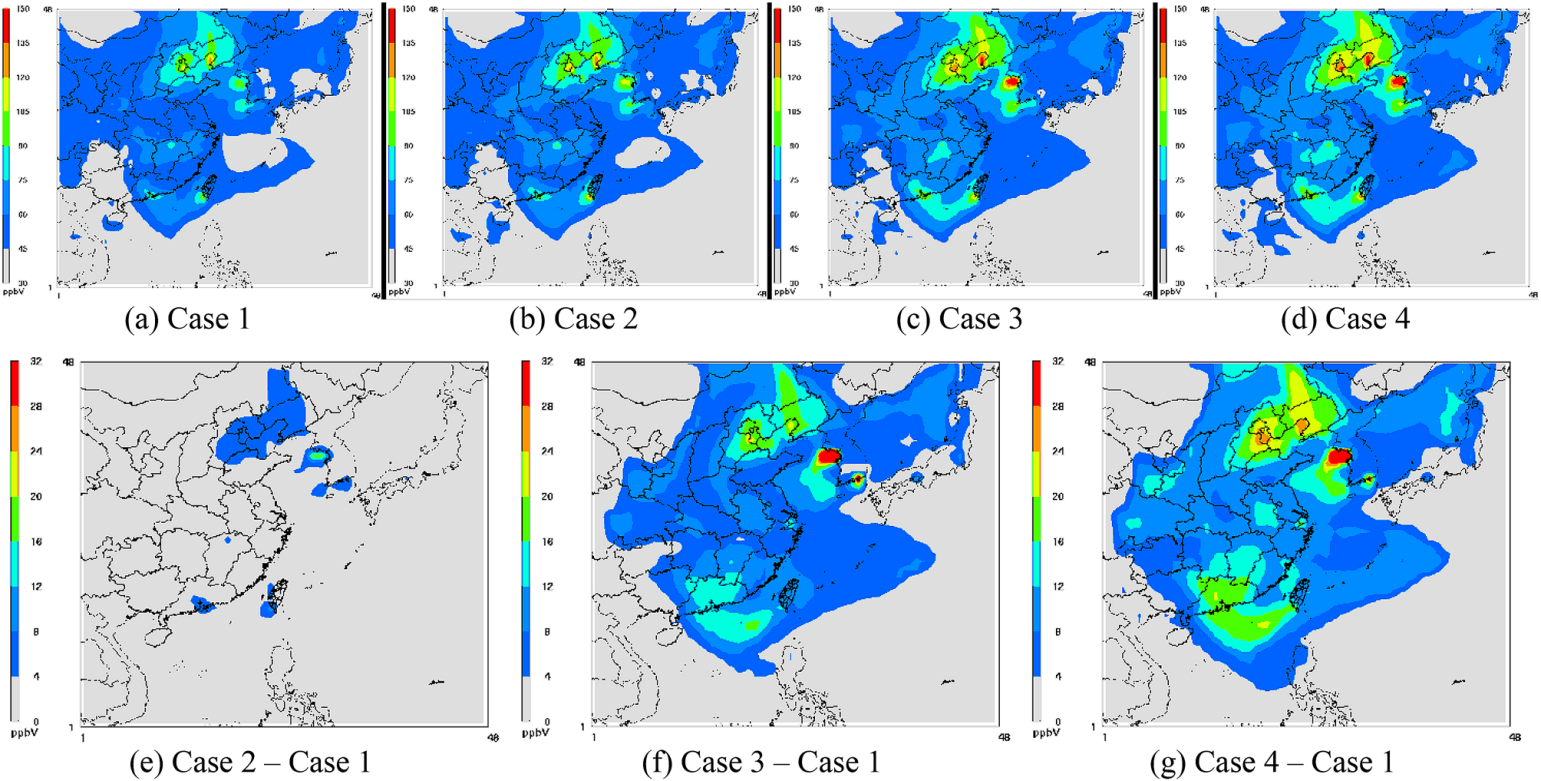
Isopleths of the O_3_ level at 2 pm on May 27, 2003, in Domain 1 [the figures are produced with Package for Analysis and Visualization of Environmental data (PAVE) v2.3.2, http://paved.sourceforge.net/].

**Figure 5:**
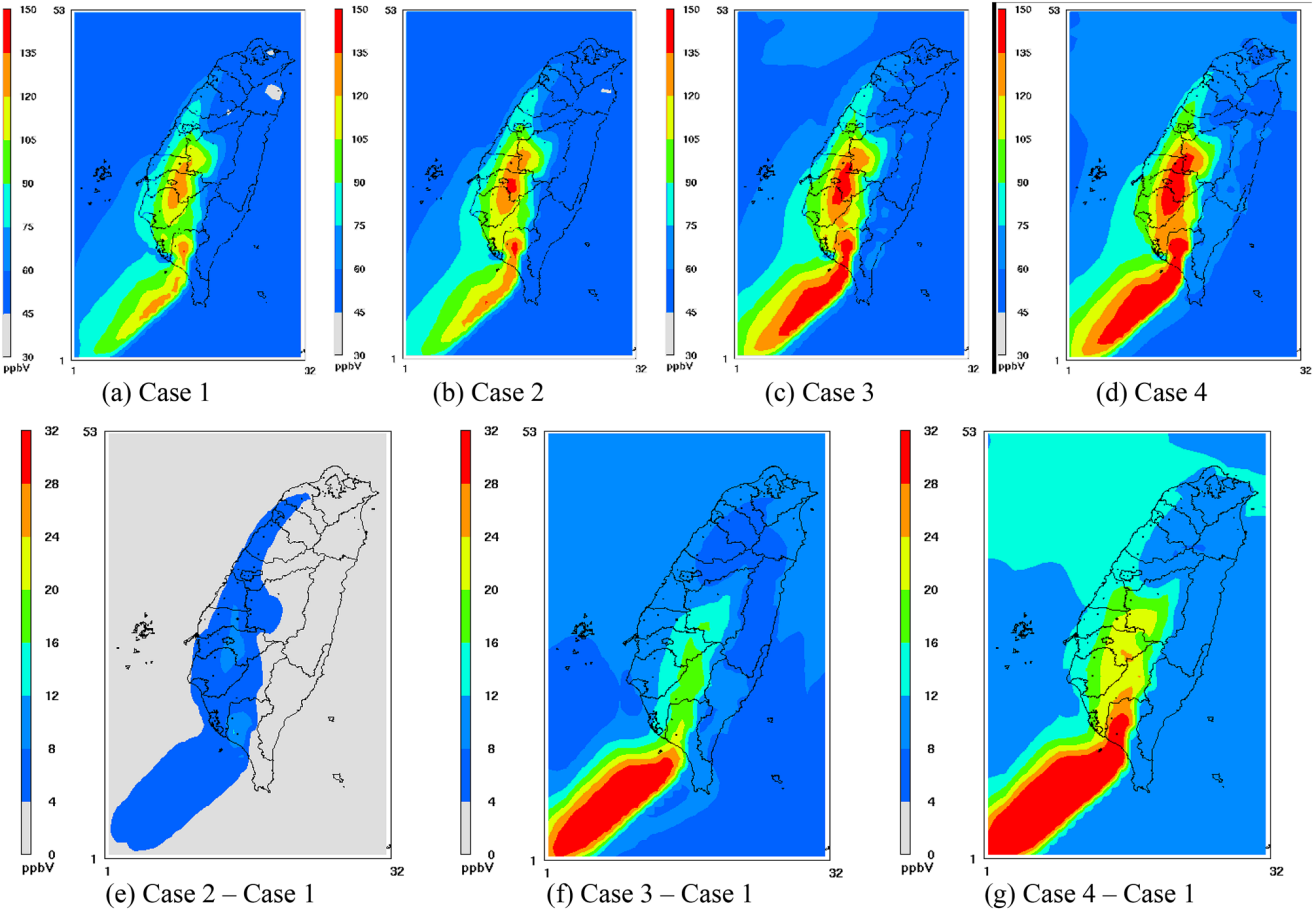
Isopleths of the O_3_ level at 2 pm on May 27, 2003, in Domain 3 [the figures are produced with Package for Analysis and Visualization of Environmental data (PAVE) v2.3.2, http://paved.sourceforge.net/].

To understand the difference between RADM2 and RACM with and without the updated photolysis rates on ozone concentration, the simulated O_3_ isopleth plots are shown in Figs. 4 and 5 for domains 1 and 3, respectively. Note that all of the ozone concentration distribution charts (Figs. 4, 5) were the simulation results at 2 pm on May 27, 2003 because the peak O_3_ levels typically occur at that time for most of the monitoring stations shown in Fig. 3. Additionally, to better illustrate the comparison between cases, the isopleths are also shown as Case x–Case 1 to highlight the net effect of Case x since Case 1 is the reference case.

For domain 1, isopleth plots for Case 1 (Fig. 4a) indicate the areas with high ozone concentrations of more than 100 ppb, including Beijing, Korea and the southwestern coast of Taiwan. The results of Case 3 (Fig. 4c) indicate that high ozone distribution areas were similar to those in Case 1; however, areas with high O_3_ concentrations expanded. The largest difference in ozone concentration (Fig. 4f) was over 30 ppb (from 105 to 135 ppb), occurring along the coastal areas in Korea.

For domain 3, the areas with high ozone concentrations of over 120 ppb in Case 1 were mainly in central and southwestern Taiwan, as well as the sea close to southern Taiwan (Fig. 5a). The largest difference in ozone concentration was greater than 30 ppb, from 120 to 150 ppb (Fig. 5f), and occurred over the sea, close to southern Taiwan. An increase of approximately 20 ppb, representing approximately 17% of ozone concentrations, occurred in southern Taiwan when RADM2 was replaced with RACM (Fig. 5f).

### Influence of the updated photolysis rate constants (Case 2 vs. Case 1)

The distributed areas with high ozone levels in Case 2 were similar to those in Case 1 in Domain 1 (Fig. 4,b, respectively). However, the areas with ozone concentrations higher than 120 ppb were larger in Case 2 than those in Case 1. The largest simulated ozone increase of 18 ppb (from 100 to 118 ppb) was observed in coastal areas around Korea, followed by southern Taiwan, where the ozone concentration increased by 10 ppb (from 106 to 116 ppb). Beijing also exhibited an increase of 7 ppb O_3_ (from 108 to 115 ppb). The influence of the ozone concentration obtained with the updated photolysis rate constants in most other areas was less than 4 ppb. Overall, the effect of the photolysis rate on the model simulations was notable.

Spatial distribution differences in the ozone concentration in Domain 3 before and after the updated photolytic rate constants were applied are shown in Fig. 5a,b, respectively. Southwestern areas of Taiwan attained high ozone concentrations (Case 1), and the areas with high ozone concentrations tended to expand after the updated data were applied. Western parts of Taiwan revealed the largest difference in the ozone concentration before and after the data update. An increase of 12 ppb in the ozone level in Case 1 over Case 2 (from 120 to 132 ppb, an increase of approximately 10%) occurred in southwestern districts of Taiwan after updating the photolysis rate constants (Fig. 5e).

### Overall comparison (Case 4 vs. Case 1)

The influence of the updated photolysis rates on RACM in Domains 1 and 3 is shown in Figs. 4g and 5g, respectively. Again, the areas with high ozone concentrations greatly expanded in Case 4 for many districts in Domain 1, including Guangdong and Taiwan. Districts with the largest concentration differences between these two cases occurred in the Beijing, Shenyang and Korean coastal areas. An ozone concentration difference of approximately 32 ppb occurred in coastal areas around Korea (Fig. 4g) when RADM2 was replaced with RACM based on the updated photolysis rates.

Spatial distribution differences in the ozone concentration in Domain 3 are shown in Fig. 5g. The ozone concentration across Taiwan increased by more than 8 ppb when RACM replaced the RADM2 mechanism with the new photolysis rates. In Taiwan, the largest concentration difference between Cases 1 and 4 occurred in southwestern Taiwan, where an ozone concentration difference of 30 ppb (approximately 25%) was found.

In general, the increased O_3_ levels obtained with RACM and the updated photolysis rate constants were higher than those reported in the literature based on alternate chemical mechanisms. For example, Jimenez et al.^3^ compared seven chemical mechanisms, including RADM2 and RACM, with a box model under various remote troposphere simulation scenarios. The difference between RADM2 and RACM in regard to O_3_ was approximately 20 ppb during high-photochemical activity hours. However, Haas et al.^48^ compared RADM2 and RACM through the use of a regional air chemistry model, MCCM, and found that the difference in O_3_ was rather small, where the variation in the correlation results was within 2%. They concluded that the RADM2 mechanism remained a reasonable alternative for consideration in simulations of regional O_3_ episodes, daily O_3_ forecasts or long-term air chemistry. To study the impact of lumped chemical mechanisms in air quality models, Arteta et al.^49^ compared a simplified mechanism (CV-MOCA2.2) and a detailed mechanism (RACM), finding that the relative difference over the entire domain was only − 7% for ozone, with a difference of approximately 5–10 ppb. The lumped-molecule mechanism (RACM2) and the lumped-structure mechanism (CB05) were also compared in simulations of O_3_ over Europe with POLAIR3D^50^. They concluded that these two mechanisms provide similar results with a domain-averaged difference of only 3 ppb over a one-month simulation period in regard to the daily maximum 8-h average O_3_ concentration. Thus, when applying updated photolysis rates, the impact on the predicted O_3_ concentration is much more notable than that of the application of different models. Furthermore, the different conclusions reached in the various studies mentioned above depended on varying temporal and spatial conditions with different VOC/NOx ratios and meteorological conditions. As such, Gross and Stockwell^6^ suggested that a broad range of simulation conditions should be considered to compare mechanisms, not just a few selected scenarios.

### Impact of the model and photolysis rate constants in polluted and clean areas

**Figure 6:**
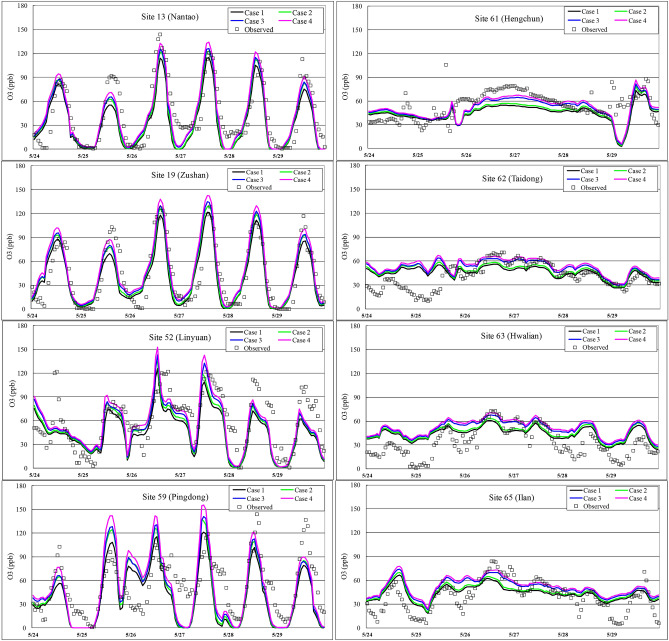
Time series of the simulated and observed ozone concentrations during the 6-d period from May 24 to 29, 2003, for Cases 1 to 4 at the stations in polluted areas (left side) and clean areas (right side).

Figure 6 shows the simulation results in all 4 cases at sites with high O_3_ levels (left column) and low O_3_ levels (right column) to juxtapose the impact of model specifications with regard to clean and polluted areas. In general, there were large differences among the simulated daily maximum ozone concentrations in these 4 cases in polluted areas. However, a limited impact was found in clean areas. It is speculated that few photochemical reactions occur in clean areas. Hence, the model with the updated photolysis rate exerts a small impact in these areas. When directly comparing RACM and RADM2 (Case 1 vs. 3), the simulated O_3_ levels are slightly higher than the observed values, regardless of the O_3_ levels at the monitoring stations. With the use of different chemical mechanisms (RACM, CB05, etc.), Chen et al.^24^ also concluded that mechanistic details are less important in polluted areas. They reasoned that under polluted conditions, reactive nitrogen chemistry dominates. In contrast, Gross and Stockwell^6^ reported that the differences in O_3_ precursors between the EMEP, RADM2 and RACM mechanisms were rather small under clean conditions and more notable under polluted conditions. The exact reasons for the observed model performance variations between clean and polluted areas remain unclear and require further evaluation.

### ***Simulated O***_***3***_*** and API and LIM levels at one station***

**Figure 7:**
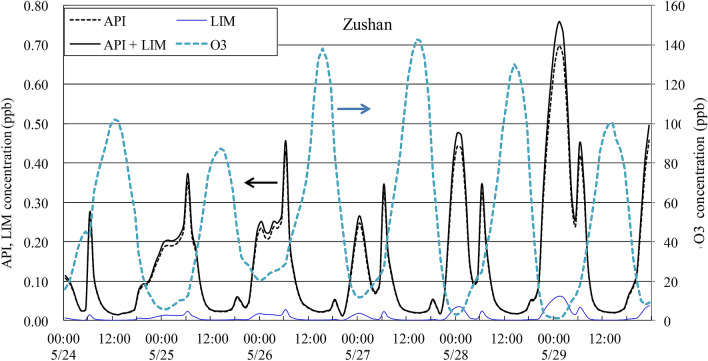
Time series of the simulated API, LIM and O_3_ concentrations during the 6-d period from May 24 to 29, 2003, at Zushan.

Since Case 4 was found to best represent the O_3_ levels in Taiwan, this case was applied to simulate two important biogenic species, as shown in Fig. 7. Simulated O_3_ concentrations are also shown for comparison. Several points must be addressed. First, the peak O_3_ concentrations are extremely high at this remote station, which is close to a forestland area. Apparently, biogenic VOCs play an important role in the interaction with O_3_, which is reflected by the clear lag between the peak concentrations of O_3_ and API. Second, the magnitude of the API values at negligible LIM levels is in agreement with those reported by other researchers (e.g., 0.4–0.6 ppb measured 2.9 m above the canopy in a spruce forest^51^), but higher simulated API values were obtained than those measured at a boreal site (peak < 0.2 ppb) in Finland^52^. These insignificant LIM levels may be due to its high reaction affinity for O_3_ to produce secondary organic aerosols (SOAs)^53^. Third, the peak concentrations occurred in the early morning and around midnight. Forkel et al.^51^ also reported that degradation of biogenic VOCs, including API and LIM, mostly occurred during the daytime via NO_3_ radical reactions, which explains the observed peak in the early morning and nighttime. Prediction of these biogenic VOCs is useful to further evaluate their impact on O_3_ formation as well as SOA production.

## Conclusions

Both RACM and RADM2 with and without updated photolysis rate constants were applied in the CMAQ model in this study to evaluate the impact of photolysis rate constants on the simulated O_3_ level. The peaks of the simulated ozone concentration during the daytime are mainly affected by the updated photolysis rate constants, while RACM results in an increase in the ozone concentration during all periods, including high ozone concentrations found at night. According to the model comparison results, the geographic location exhibiting the largest difference in the maximum ozone concentration occurred in southwestern Taiwan. Application of the updated photolysis rate constants in RADM2 resulted in an increase of 12 ppb (10%) in the maximum ozone concentration in southwestern Taiwan, while the use of RACM without the updated photolysis rates resulted in an increase of 20 ppb (17%) in the maximum ozone concentration in the same area. When RACM was combined with the updated photolysis rate constants, the difference in the maximum ozone concentration reached 30 ppb (25%). The application of both the updated photolysis rate constants and RACM instead of RADM2 improved all three error analysis indicators (UPPA, MNBR and MANGE) of the simulation results over real observed data.

## Supplementary Information

Supplementary Information.

## References

[1] Stockwell WR, Kirchner F, Kuhn M, Seefeld S (1997). A new mechanism for regional atmospheric chemistry modeling. J. Geophys. Res..

[2] Marvin MR (2017). Impact of evolving isoprene mechanisms on simulated formaldehyde: An inter-comparison supported by in situ observations from SENEX. Atmos. Environ..

[3] Jimenez P, Baldasano JM, Dabdub D (2003). Comparison of photochemical mechanisms for air quality modeling. Atmos. Environ..

[4] Stockwell WR, Middleton P, Chang JS, Tang X (1990). The second generation regional Acid Deposition Model chemical mechanism for regional air quality modeling. J. Geophys. Res..

[5] Goliff WS, Stockwell WR, Lawson CV (2013). The regional atmospheric chemistry mechanism, version 2. Atmos. Environ..

[6] Gross A, Stockwell WR (2003). Comparison of the EMEP, RADM2 and RACM mechanisms. J. Atmos. Chem..

[7] Luecken DJ, Phillips S, Sarwar G, Jang C (2008). Effects of using the CB05 vs. SAPRC99 vs. CB4 chemical mechanism on model predictions: Ozone and gas-phase photochemical precursor concentrations. Atmos. Environ..

[8] Yu S (2010). Eta-CMAQ air quality forecasts for O3 and related species using three different photochemical mechanisms (CB4, CB05, SAPRC-99): Comparisons with measurements during the 2004 ICARTT study. Atmos. Chem. Phys..

[9] Ying Q, Li J (2011). Implementation and initial application of the near-explicit master chemical mechanism in the 3D Community Multiscale Air Quality (CMAQ) model. Atmos. Environ..

[10] Simpson D, Olendrzynski K, Semb A, Støren E, Unger S (1997). Photochemical Oxidant Modelling in Europe: Multi-Annual Modelling and Source-Receptor Relationships, EMEP MSCW Report 3/97.

[11] Yarwood, G., Rao, S., Yocke, M., & Whitten, G. Z. *Updates to the Carbon Bond chemical mechanism: CB05. Final Report to the US EPA, RT-04006752005)*. http://www.camx.com/publ/pdfs/CB05_Final_Report_120805.pdf.

[12] Carter, W. P. L. *Implementation of the SAPRC-99 Chemical Mechanism into the Models-3 FRAMEWORK. Report to the United States Environmental Protection Agency* (2000). http://www.cert.ucr.edu/~carter/absts.htm#s99mod3.

[13] Gery MW, Whitten GZ, Killus JP, Dodge MC (1989). A photochemical kinetics mechanism for urban and regional scale computer modeling. J. Geophy. Res..

[14] Chen S (2010). A comparison of chemical mechanisms based on TRAMP-2006 field data. Atmos. Environ..

[15] Sarwar G (2013). A comparison of atmospheric composition using the carbon bond and regional atmospheric chemistry mechanisms. Atmos. Chem. Phys..

[16] Byun DW, Ching JKS, USEPA (1999). Science Algorithms of the EPA Models-3 Community Multiscale Air Quality (CMAQ) Modeling System EPA/600/R99/030.

[17] Seinfeld JH, Pandis SN (2006). Atmospheric Chemistry and Physics: from Air Pollution to Climate Change.

[18] Epstein SA, Tapavicza E, Furche F, Nizkorodov SA (2013). Direct photolysis of carbonyl compounds dissolved in cloud and fog droplets. Atmos. Chem. Phys..

[19] Monks PS (2005). Gas-phase radical chemistry in the troposphere. Chem. Soc. Rev..

[20] Helmiga D, Bocqueta F, Cohena L, Oltmans SJ (2007). Ozone uptake to the polar snowpack at Summit, Greenland. Atmos. Environ..

[21] Hembeck L (2019). Measured and modelled ozone photochemical production in the Baltimore Washington airshed. Atmos. Environ. X..

[22] Alicke B, Platt U, Stutz J (2002). Impact of nitrous acid photolysis on the total hydroxyl radical budget during the Limitation of Oxidant Production/Pianura Padana Produzione di Ozono study in Milan. J. Geophys. Res..

[23] Frost GJ, Ellison GB, Vaida V (1999). Organic peroxyl radical photolysis in the near-infrared: effects on tropospheric chemistry. J. Phys. Chem. A.

[24] Chen S, Brune WH (2012). Global sensitivity analysis of ozone production and O3–NOx–VOC limitation based on field data. Atmos. Environ..

[25] Hanna SR (2001). Uncertainties in predicted ozone concentrations due to input uncertainties for the UAM-V photochemical grid model applied to the July 1995 OTAG domain. Atmos. Environ..

[26] He S, Carmichael GR (1999). Sensitivity of photolysis rates and ozone production in the troposphere to aerosol properties. J. Geophy. Res..

[27] Wang W (2019). The impact of aerosols on photolysis frequencies and ozone production in Beijing during the 4-year period 2012–2015. Atmos. Chem. Phys..

[28] Grell, G. A., Dudhia, J., & Stauffer, D. R. *A Description of the Fifth-Generation Penn State/NCAR Mesoscale Model (MM5). NCAR Tech. Note, NCAR/TN-398þSTR*. (National Center for Atmospheric Research, Boulder, CO, 1995).

[29] Chen TF, Chang KH, Tsai CY (2014). Modeling direct and indirect effect of long range transport on atmospheric PM2.5 levels. Atmos. Environ..

[30] Chen TF, Chang KH, Lee TH (2019). Simulation and analysis of causes of a haze episode by combining CMAQ-IPR and brute force source *s*ensitivity method. Atmos. Environ..

[31] Chen TF, Chang KH, Tsai CY (2019). A modeling study of assessment of the effectiveness of combining foreign and local emission control strategies. Atmos. Res..

[32] Chen TF, Chang KH, Tsai CY (2017). Modeling approach for emissions reduction of primary PM2.5 and secondary PM2.5 precursors to achieve the air quality target. Atmos. Res..

[33] JPL. *Chemical Kinetics and Photochemical Data for Use in Atmospheric Studies*. *Evaluation Number 15* (Jet Propulsion Laboratory, NASA, Pasadena, California, 2006).

[34] Lin CY (2008). Numerical study of the impact of urbanization on the precipitation over Taiwan. Atmos. Environ..

[35] Chien FC, Liu YC, Jou BJD (2006). MM5 ensemble mean forecasts in the Taiwan area for the 2003 Mei-Yu Season. Weather Forecast..

[36] Holnicki P, Nahorski Z (2015). Emission data uncertainty in urban air quality modelling: Case study. Environ. Model. Assess..

[37] Taiwan EPA. *Update and Management of Air Pollution Emission Inventory and Air Pollution Degradation Estimation. Final Rep*. (CTCI, Taipei, Taiwan, 2006) (**in Chinese**).

[38] Chang KH, Yu JY, Chen TF, Lin YP (2009). Estimating Taiwan biogenic VOC emission: Leaf energy balance consideration. Atmos. Environ..

[39] Tsai CY, Chen TF, Lin YP, Chang KH (2020). Air quality modeling: Effect of land use database using remote sensing data. J. Innov. Technol..

[40] Ohara T (2007). An Asian emission inventory of anthropogenic emission sources for the period 1980–2020. Atmos. Chem. Phys..

[41] Chen TF, Chen CH, Yu JY, Lin YB, Chang KH (2020). Estimation of biogenic VOC emissions in East Asia with new emission factors and leaf energy balance considerations. J. Innov. Technol..

[42] Brown SS (2006). Variability in nocturnal nitrogen oxide processing and its role in regional air quality. Science.

[43] Singh HB (2007). Reactive nitrogen distribution and partitioning in the North American troposphere and lowermost stratosphere. J. Geophys. Res..

[44] Epa US (2008). Air Quality Modeling Platform for the Ozone National Ambient Air Quality Standard: Final Rule Regulatory Impact Analysis.

[45] Emery, C. A., Tai, E., & Yarwood, G. *Enhanced Meteorological Modeling and Performance Evaluation for Two Texas Ozone Episodes*. Prepared for The Texas Natural Resource Conservation Commission, ENVIRON International, Novato, CA (2001).

[46] Russell R, Dennis R (2000). NARSTO critical review of photochemical models and modeling. Atmos. Environ..

[47] Chang KH (2008). Modeling approach for emission reduction of O_3_ precursors in Southern Taiwan. Atmos. Environ..

[48] Haas E, Forkel R, Suppan P (2007). Application and intercomparison of the RADM2 and RACM atmospheric chemistry mechanism including a new isoprene degradation scheme within the online-coupled regional meteorology chemistry model MCCM. Int. J. Environ. Pollut..

[49] Arteta J, Cautenet S, Taghavi M, Audiffren N (2006). Impact of two chemistry mechanisms fully coupled with mesoscale model on the atmospheric pollutants distribution. Atmos. Environ..

[50] Kim Y, Sartelet K, Seigneur C (2009). Comparison of two gas-phase chemical kinetic mechanisms of ozone formation over Europe. J. Atmos. Chem..

[51] Forkel R (2006). Trace gas exchange and gas phase chemistry in a Norway spruce forest: A study with a coupled 1-dimensional canopy atmospheric chemistry emission model. Atmos. Environ..

[52] Hakola H, Laurila T, Rinne J, Puhto K (2000). The ambient concentrations of biogenic hydrocarbons at a northern European, boreal site. Atmos. Environ..

[53] Jonsson AM, Hallquist M, Ljungström E (2006). Impact of humidity on the ozone initiated oxidation of limonene, δ^3^-carene, and α-pinene. Environ. Sci. Technol..

[54] Bass AM, Ledford AE, Laugfer AH (1976). Extinction coefficients of and N_2_O_4_. J. Res. Natl. Bur. Stand. A.

[55] Gardner EP, Wijayaratne RD, Calvert JG (1984). Primary quantum yields of photodecomposition of acetone in air under tropospheric conditions. J. Phys. Chem..

[56] Troe JZ (2000). Are primary quantum yields of NO2 photolysis at λ ≤ 398 nm smaller than unity?. J. Phys. Chem..

[57] JPL. *Chemical Kinetics and Photochemical Data for Use in Stratospheric Modeling. Evaluation Number 8* (Jet Propulsion Laboratory, NASA, Pasadena, California, 1987).

[58] Moortgat GK, Kudzus E (1978). Mathematical expression for the O(^1^D) quantum yields from O3 photolysis as a function of temperature (230–320 K) and wavelength (298–320 nm). Geophys. Res. Lett..

[59] JPL. *Chemical Kinetics and Photochemical Data for Use in Stratospheric Modeling. Evaluation Number 11* (Jet Propulsion Laboratory, NASA, Pasadena, California, 1994).

[60] Stockwell WR, Calvert JG (1978). The near ultraviolet spectrum of gaseous HONO and N_2_O_3_. J. Photochem..

[61] Molina LT, Molina MJ (1981). UV absorption cross sections of HO2NO2 vapor. J. Photochem..

[62] Graham RA, Johnson HS (1978). The photochemistry of NO_3_ and the kinetics of the N_2_O_5_-O_3_ system. J. Phys. Chem..

[63] Magnotta F, Johnson HS (1980). Photodissociation quantum yields for the NO_3_ free radical. Geophys. Res. Lett..

[64] Johnston HS, Davis HF, Lee YT (1996). NO_3_ photolysis product channels: Quantum yields from observed thresholds. J. Phys. Chem..

[65] Lin CL, Rohatgi NK, DeMore WB (1978). Ultraviolet absorption cross sections of hydrogen peroxide. Geophys. Res. Lett..

[66] Moortgat, G. K. *et al. Laboratory Measurement of Photolytic Parameters for Formaldehyde. Final Rep. FAA-EE-80-47* (Office of Environment and Energy, Federal Aviation Administration, US Dept. of Transportation, Washington, D.C., 1980).

[67] Moortgat GK, Seiler W, Warneck P (1983). Photodissociation of HCHO in air: CO and H2 quantum yields at 220 and 330 K. J. Phys. Chem..

[68] Smith GD, Molina LT, Molina MJ (2002). Measurement of radical quantum yields from formaldehyde photolysis between 269 and 339 nm. J. Phys. Chem. A.

[69] Calvert JG, Pitts JN (1966). Photochemistry.

[70] Meyrahn H, Moortgat GK, Warneck P, Herbert F (1982). The Photolysis of acetaldehyde under atmospheric conditions. Atmospheric Trace Constituents.

[71] Atkinson R (1997). Evaluated kinetic, photochemical and heterogeneous data for atmospheric chemistry: Supplement V. IUPAC Subcommittee on gas kinetic data evaluation for atmospheric chemistry. J. Phys. Chem. Ref. Data.

[72] Molina LT, Arguello G (1979). Ultraviolet absorption spectrum of methylhydroperoxide vapor. Geophys. Res. Lett..

[73] Giguere PA, Olmos AW (1956). Sur le spectre ultraviolet de l’acide peracétique et l’hydrolyse des peracétates. Can. J. Chem..

[74] Martinez RD (1992). The near UV absorption spectra of several aliphatic aldehydes and ketones. Atmos. Environ..

[75] Plum CN (1983). OH radical rate constants and photolysis rates of α dicarbonyls. Environ. Sci. Technol..

[76] Carter WPL, Atkinson R (1989). Alkyl nitrate formation from the atmospheric photooxidation of alkanes: A revised estimation method. J. Atmos. Chem..

[77] Atkinson R (1992). Evaluated kinetic and photochemical data for atmospheric chemistry: Supplement IV. J. Phys. Chem. Ref. Data.

[78] Gierczak T (1997). Atmospheric fate of methyl vinyl ketone and methacrolein. J. Photochem. Photobiol. A.

