# Supplementary material for: Comparison of the RADM2 and RACM chemical mechanisms in O3 simulations: effect of the photolysis rate constant
Source: Sci Rep. 2021 Mar 3;11:5024. doi: 10.1038/s41598-021-84629-4 (PMC7930097; doi:10.1038/s41598-021-84629-4)
Supplement: Supplementary file 1 — Supplementary Information. [file 41598_2021_84629_MOESM1_ESM.docx]

Comparison of the RADM2 and RACM chemical mechanisms

in O_3_ simulations: Effect of the photolysis rate constant

Chien-Hung Chen^1^, Tu-Fu Chen^2^, Shang-Ping Huang^2^, Ken-Hui Chang^2,*^,

^1^Graduate school of Engineering Science and Technology, National Yunlin University of Science and Technology, Yunlin, Taiwan.

^2^Department of Safety, Health and Environmental Engineering, National Yunlin University of Science and Technology, Yunlin, Taiwan.

Supplementary Material

Figure SM-1 Comparison of original and adjusted diurnal variations of NO_2_ photolysis rate

Figure SM-2 Comparison of original and adjusted diurnal variations of O_3_ photolysis rate

Figure SM-3 Comparison of original and adjusted diurnal variations of HONO photolysis rate

Figure SM-4 Comparison of original and adjusted diurnal variations of HNO_3_ photolysis rate

Figure SM-5 Comparison of original and adjusted diurnal variations of HNO_4_ photolysis rate

Figure SM-6 Comparison of original and adjusted diurnal variations of NO_3_ photolysis rate


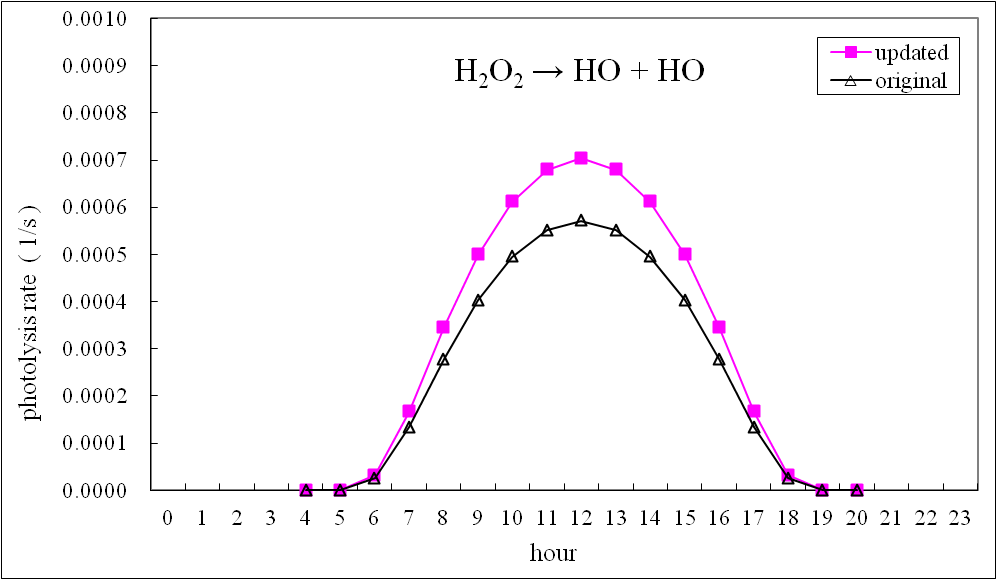


Figure SM-7 Comparison of original and adjusted diurnal variations of H_2_O_2_ photolysis rate
